# Sexual orientation based health disparities in Chile

**DOI:** 10.1371/journal.pone.0296923

**Published:** 2024-01-25

**Authors:** Laura Nettuno, Samuel Mann, Gilbert Gonzales

**Affiliations:** 1 Department of Economics, Vanderbilt University, Nashville, Tennessee, United States of America; 2 RAND Corporation, Arlington, Virginia, United States of America; 3 Department of Medicine, Health & Society, Department of Health Policy, and Program for Public Policy Studies, Vanderbilt University, Nashville, Tennessee, United States of America; Bard College, UNITED STATES

## Abstract

Numerous studies from Europe and North America have documented sexual orientation-based health disparities, but due to data limitations, very little is known about the health of sexual minorities (i.e., lesbians, gay men, bisexual individuals, and other non-heterosexual populations) in developing countries. This research note uses newly available nationally representative data from the Chilean Socio-Economic Characterization Survey (CASEN) to explore sexual orientation-based disparities in self-rated health, health insurance coverage, and healthcare utilization in Chile. Our findings indicate that sexual minority respondents report worse self-rated health and greater health care utilization, and that sexual minority men are more likely to have private health insurance relative to heterosexual men. These findings are important in facilitating continued efforts to reduce health disparities in Latin America.

## Introduction

A growing body of population-based research has described and identified socioeconomic and health disparities for sexual minorities. Prior evidence suggests that structural and interpersonal discrimination and stigma against sexual minorities can exacerbate health disparities through minority stress processes [1]. Indeed, numerous studies from Europe and North America have demonstrated that sexual minorities are more likely to report worse health, have greater psychological distress, report greater rates of adverse health behaviors such as smoking and alcohol consumption, and are more likely to be uninsured and face barriers to care than their heterosexual counterparts [1–12]. Existing studies have relied heavily on self-reported survey data from the United States, Canada, and the United Kingdom but, as highlighted by the Inter-American Development Bank [13] little is known regarding the health of sexual minorities outside of the European and US contexts, and there is a distinct lack of research on sexual minorities in Latin America and the Caribbean, largely due to data limitations. Of the available research, very recent studies using relatively small or convenience samples suggest that sexual minorities in Brazil [14], Chile [15], and Mexico [16] may also report adverse health outcomes and barriers to care.

This study leverages newly available data from Chile to examine health insurance, self-reported health, and healthcare utilization disparities between self-disclosed sexual minorities and their heterosexual peers. In doing so, this study provides evidence of sexual minority health disparities in Chile using large-scale representative data and provides new insights into the health, health insurance status, and healthcare utilization of sexual minorities in Latin America which will be important in facilitating continued efforts to reduce health disparities within this region.

## The Chilean sexual minority population

Recent estimates from the OECD indicate that around 1–2% of the Chilean population identifies as lesbian, gay, or bisexual [17, 18], which given the population size of 19.5 million people translates to around 200,000–350,000 people. Sexual minority Chileans live in the bottom tier of OECD countries in terms of a country’s inclusivity towards lesbian, gay, bisexual, transgender and queer (LGBTQ+) populations, according to the OECD [19]. Nonetheless, Chile has witnessed significant LGBTQ+ progress in recent years with the introduction of anti-discrimination laws in 2012, the legalization of same-sex marriage in 2022, and broad improvements in attitudes towards sexual minorities [18]. Compared to peer nations in Latin America and the Caribbean, Chileans are relatively more accepting of LGBTQ+ populations and identities [20].

While there has been legislative and social progress for LGBTQ+ people in Chile, there remains a dearth of research on the health of sexual minorities, largely due to data limitations. However, a handful of recent studies have begun to document the health of sexual minorities in Chile. Barrientos et al. [21] for example, found that 9% of gay men and 12% of lesbians scored above the cut-off point for anxiety or depression in their analysis of 447 sexual minorities from four Chilean cities. Building on this, research has demonstrated that these high rates of poor health among sexual minorities in Chile were exacerbated during the COVID-19 pandemic [22, 23], and recent analyses have demonstrated that the mental health of sexual minorities in Chile is associated with both internalized and experienced stigma [24]. While informative, these studies rely on convenience or snowball samples, with no heterosexual comparison group. Further, these studies are non-representative with small sample sizes, giving doubt to the generalizability of findings.

## Data & methods

### Data

To document sexual minority health disparities in Chile we make use of the large, nationally representative, Chilean National Socio-Economic Characterization Survey (CASEN). Although the CASEN is primarily a social development-focused household survey that aims to understand poverty and the socioeconomic situation of Chilean families, we leverage the detailed individual demographic and health information in the CASEN to study disparities between sexual minorities and heterosexual respondents. Data collection is conducted via face-to-face interviews using a household roster format. One adult is selected as the designated informant for the household. Survey administrators are instructed to ask to speak to the head of household first. If the head of household is unavailable at the time of the interview, another available adult who resides in the household is selected as the designated informant. Data are collected from members of the household who are present at the time of the interview or through the household informant for those who are not present.

### Sexual identity

In 2015, the Ministry of Social Development added a new module to the survey, which allowed adult survey respondents who were present at the time of the interview to identify their sexual orientation (and, later in 2017, gender minority status). This paper focuses on sexual orientation but we control for gender minority status to account for any health effects of being non-cisgender (our findings are not sensitive to controlling for gender minority status). To elicit accurate information about sexual and gender identity, survey administrators are instructed to only ask *adults who are present at the time of the interview* to self-identify their own sexual orientation and gender identity. As such, our sample includes all adults over the age of 18 years to study the relationship between sexual identity and health. For those who are not present, information regarding sexual orientation and gender identity is missing. That is, sexual and gender identity cannot be third-party reported. Because the sexual orientation of absent individuals is unknown, they are not included in the sample for this analysis.

Survey administrators define the term “sexual orientation” before asking the respondent about their sexual minority status. Specifically, respondents are told the following: “Next, I am going to ask you some questions related to sexual orientation and gender identity. Your answers will be confidential and used only for statistical purposes. ‘Sexual orientation’ is understood as the attraction that a person may have towards the opposite sex (heterosexual), the same sex (homosexual), or towards both (bisexual).” Survey respondents are also presented with a visual aid to help explain sexual orientation, shown in S1 Fig. Respondents are then asked: “Which of these alternatives best defines your sexual orientation?” The response options given are: “heterosexual”; “gay/lesbian”; “bisexual”; “something else”; and “I don’t know.” To increase power and precision we combine LGB (lesbian, gay, and bisexual) responses into a single indicator, denoted as *SexualMinority*_*i*_ in Eq (1) below. We include separate controls for those individuals who reported “something else” not listed in the terms offered. We also control separately for those who report that they “don’t know” which term best describes them. Around 1% of the sample self-identified as lesbian, gay, or bisexual which is similar to prior estimates of the size of the LGB population in Chile from the OECD [17, 18].

### Outcomes

The CASEN includes information on health insurance, self-rated health, and healthcare utilization, and we use each of these to document an overview of sexual minority health disparities in Chile. To estimate sexual minority health insurance disparities, we consider two binary variables: first, whether the respondent reports that they are uninsured, and second, whether the respondent reports that they have private health insurance (S1 File provides further details regarding the Chilean health care and insurance system). Self-reported health measures come from a self-rated overall health score ranging from 1 (very poor) to 7 (excellent), and self-reports of common health conditions. We use these data to create two binary variables, one that takes the value 1 if the respondent reports very good or excellent health and zero otherwise, and another that takes the value 1 if the respondent reports being treated for a common health condition in the last 12 months and zero otherwise. Each of these conditions are listed in S2 File. In S1 Table, we estimate the association between being a sexual minority and each individual condition. To study healthcare utilization, we use data from a question that asks respondents the number of doctor visits they have had in the prior 3 months. S2 File provides the survey questions for each outcome variable.

### Methods

To estimate the relationship between sexual minority status and health outcomes, we estimate the following model for men and women separately:

$$Y_{i}=\;\beta_{0}+\;\beta_{1}X_{i}+\;\beta_{2}\left(SexualMinority_{i}\right)+\;\epsilon_{i}$$ (1)

where *Y* represents the outcome of interest for individual *i*. The coefficient of interest is β_2_, which measures the association between sexual minority status and the health outcome of interest. *X*_*i*_ is a vector of control variables that have been shown to be associated with health-related outcomes. We include controls for: age and age-squared, gender minority status, urbanicity, indigenous identity, immigrant status, whether the respondent is employed, dummies for education (no schooling, less than high school, some college, bachelor’s degree, or graduate school with the omitted category being high school education), dummies for marital status (married, partnered, widowed, divorced—omitted category is single), the number of adults in the household, and the number of children in the household. We also control for survey wave and region of residence. In all models, we use the survey weights provided by CASEN (Although the survey weights do not take sexual orientation and gender identity into account, using the CASEN weights helps to improve population representativeness of the sample on other dimensions All results presented below are robust to estimating unweighted models) and estimate White standard errors robust to heteroskedasticity.

## Results

### Descriptive statistics

Table 1 presents descriptive statistics from the CASEN survey data. We present sample averages for sexual minority men in column 1; heterosexual men in column 2; sexual minority women in column 3; and heterosexual women in column 4. We present information on our control variables and key outcome measures.

**Table 1:** Descriptive statistics.

	(1)	(2)	(3)	(4)
	SM men	Heterosexual men	SM women	Heterosexual women
Age	35.87***	49.20	38.84***	49.03
	(13.79)	(17.87)	(16.69)	(17.34)
Urban	0.953*** (0.212)	0.856 (0.351)	0.908*** (0.289)	0.871 (0.335)
Indigenous	0.056** (0.23)	0.083 (0.276)	0.082 (0.274)	0.088 (0.283)
Immigrant	0.080* (0.272)	0.045 (0.207)	0.047 (0.212)	0.031 (0.174)
No schooling	0.004*** (0.066)	0.020 (0.138)	0.014 (0.119)	0.022 (0.147)
Less than HS	0.129*** (0.336)	0.353 (0.478)	0.234*** (0.424)	0.401 (0.490)
High school	0.202*** (0.401)	0.299 (0.458)	0.273* (0.446)	0.314 (0.464)
Some college	0.213*** (0.410)	0.107 (0.309)	0.208*** (0.406)	0.081 (0.273)
Bachelors	0.340*** (0.474)	0.195 (0.396)	0.200* (0.400)	0.166 (0.372)
Graduate	0.112** (0.316)	0.026 (0.159)	0.072 (0.258)	0.016 (0.124)
Married	0.100*** (0.300)	0.430 (0.495)	0.236*** (0.425)	0.368 (0.482)
Partnered	0.240** (0.427)	0.184 (0.387)	0.254*** (0.435)	0.168 (0.374)
Widowed	0.014*** (0.119)	0.042 (0.200)	0.038*** (0.191)	0.102 (0.302)
Divorced	0.032*** (0.175)	0.082 (0.274)	0.051*** (0.220)	0.119 (0.323)
# of HH adults	2.095*** (1.044)	2.495 (1.142)	2.352*** (1.108)	2.527 (1.157)
Pr(any children in the house)	0.142*** (0.349)	0.373 (0.484)	0.355*** (0.479)	0.514 (0.500)
Private insurance	0.411*** (0.492)	0.191 (0.393)	0.185* (0.389)	0.118 (0.323)
Uninsured	0.044 (0.204)	0.039 (0.194)	0.049*** (0.217)	0.022 (0.146)
Health score ≥ 5	0.895*** (0.306)	0.841 (0.365)	0.828* (0.378)	0.796 (0.403)
Pr(treated for illness in last 12 mo.)	0.270 (0.444)	0.313 (0.464)	0.307*** (0.461)	0.419 (0.493)
# primary care consultations	0.413*** (1.203)	0.304 (1.251)	0.433 (1.394)	0.431 (1.344)
N	854	67,462	1,041	122,806

Notes: Weighted means (standard deviations) for demographics and economic outcomes by sex assigned at birth and gender identity.

*, **, and *** denote statistically significant differences between columns 1 & 2 for men and columns 3 & 4 for women at 10%, 5%, and 1%, respectively.

Although the survey weights do not take sexual orientation and gender identity into account, using the CASEN person-level weights helps to improve population representativeness of the sample on other dimensions.

The demographic patterns in Table 1 largely confirm results from prior studies in economics and demography that rely on US data (e.g., Badgett, Carpenter and Sansone [25]). We find that sexual minority men and women are both younger and more highly educated than their heterosexual counterparts. Sexual minorities disproportionately live in urban areas, are less likely to be from indigenous communities (especially for men) and are more likely to be immigrants. Male and female sexual minorities are significantly less likely to be married, widowed, or divorced, but are slightly more likely to be in an unmarried couple. Sexual minorities, irrespective of sex, live in households with fewer adults, and are less likely to have children, though 35.5 percent of sexual minority women have children, which is consistent with prior work that has documented higher levels of parenthood among lesbians compared to gay men [25].

Inconsistent with evidence from the US (see for example Gonzales, Przedworski and Henning-Smith [8]) male and female sexual minorities in Chile are more likely to report very good or excellent health, report greater health care utilization (proxied by the number of doctor consultations one has received in the prior three months) and are less likely to have a common health condition. However, insurance disparities remain; female sexual minorities are around 3 percentage points more likely to be uninsured and male sexual minorities are around 0.5 percentage points more likely to be uninsured compared to their heterosexual counterparts, in unadjusted models. The smaller gap for male sexual minorities is largely driven by a significantly greater uptake of private insurance among male sexual minorities compared to heterosexual men.

### Sexual orientation based disparities in insurance, health, and utilization in Chile

Having established unadjusted differences in insurance, health status, and health care utilization across sexual orientation we next examine whether unadjusted patterns remain once observable individual-level covariates and survey wave fixed effects have been included. Table 2 presents adjusted models for men in Panel A and women in Panel B, following Eq (1). Each column refers to a different outcome: uninsured (column 1), private insurance (column 2), very good or excellent self-rated health (column 3), treated for a common health condition (column 4), and number of doctor visits (column 5).

**Table 2:** Main results.

	(1)	(2)	(3)	(4)	(5)
	Uninsured	Private Insurance	Health score ≥5	Treated for common illness in last 12 months	Number of primary care consultations in the last 3 months
**Panel A: Men**					
Sexual Minority	-0.013	0.052*	-0.027**	0.099***	0.172***
	(0.010)	(0.027)	(0.013)	(0.024)	(0.063)
Mean of outcome:	0.039	0.195	0.842	0.313	0.306
N	67,029	67,029	67,740	67,552	67,866
**Panel B: Women**					
Sexual Minority	0.019**	0.003	-0.034**	0.014	0.058
	(0.009)	(0.017)	(0.016)	(0.019)	(0.061)
Mean of outcome:	0.022	0.119	0.796	0.418	0.431
N	122,349	122,349	122,907	122,648	123,100

Notes:

*, **, and *** denote statistical significance at 10%, 5%, and 1%, respectively.

Standard errors are reported below estimates in parentheses. OLS models. Specifications control for age and its square, indigenous and immigrant status, gender minority status, education, marital status, the number of adults and number of children in the household, urbanicity, survey year, and region. Results use person-level survey weights, and standard errors are robust to heteroskedasticity.

For health insurance we document different patterns by sex. Female sexual minorities are around 2 percentage points more likely to be uninsured than their heterosexual counterparts, everything else held constant. Given a lack of data on eligibility, it is not possible to distinguish between differences in eligibility and differences in enrollment. Male sexual minorities on the other hand do not significantly differ from their heterosexual counterparts on uninsurance, largely driven by male sexual minorities being significantly more likely to have private health insurance compared to their heterosexual counterparts.

In terms of self-rated health, both male and female sexual minorities are significantly less likely to report having very good or excellent health. Male sexual minorities are around 3 percentage points less likely to report very good or excellent health, which given the baseline mean, is equivalent to a 3.2% disparity in the incidence of very good or excellent health. Additionally, male sexual minorities are significantly more likely to report treatment for a common health condition: male sexual minorities are around 9 percentage points (or 31.6%) more likely to report having a common health condition. In additional results (S1 Table) we further separate the common condition variable by the 21 different conditions that are reportable in the CASEN data. These results demonstrate that the higher incidence of common health conditions among sexual minority men is driven by a higher incidence of depression and other (non-specified) conditions. Notably, HIV/ Aids is not a specifiable common health condition.

Sexual minority women are around 3–4 percentage points less likely than their heterosexual counterparts to report very good or excellent health. Given the baseline mean this translates to a 4.3% lower likelihood of reporting very good or excellent health. Sexual minority women do not significantly differ from heterosexual women in terms of the likelihood of having a common health condition. In S2 Table we re-estimate our main models including separate variables for gay/lesbian and bisexual individuals. By splitting the already-small sexual minority sample in this way we lose statistical power, and therefore precision, making several patterns statistically insignificant. Nonetheless, these results demonstrate that the male private insurance disparity is driven by bisexual men, while the self-rated health, common condition, and health care utilization disparity is drive by gay men.

Finally, results in Table 2 document disparities in health care utilization. Male sexual minorities report a significantly greater number of doctor visits (0.17) compared to heterosexual men. Female sexual minorities do not significantly differ from heterosexual women in terms of the number of doctor visits in the prior three months.

## Discussion and conclusions

This study leverages newly available population-based data to document disparities in health insurance coverage, self-reported health, and health care utilization by sexual orientation in Chile. Our findings demonstrate that sexual minorities in Chile report significantly worse health and significantly greater health care utilization than their heterosexual counterparts.

Limitations to this study included self-reported data on sexual orientation identity and health, which may be vulnerable to social desirability bias, misclassification, and recall bias. Some participants may not be comfortable disclosing accurate sexual orientations with family members or roommates present. Also missing from the survey are unhoused individuals; discrimination and socioeconomic hardships may disproportionately affect sexual minorities that lead to increased experiences of homelessness. Thus, the disparities reported here may be underestimated based on the magnitude of unobserved or missing sexual minorities. The survey also did not ascertain information about the causes of needing treatment for specific conditions–which may result from health behaviors (e.g., tobacco use) or experiences of interpersonal and structural homophobia. An additional possible limitation is that survey methodology mechanically resulted in there existing more women in the sample than men. Generally, household division of labor norms means that women are more likely to be at home providing domestic labor when the CASEN survey administrator knocks on the door, while men are typically the primary breadwinners and are more likely to be out of the house working. Since we are forced to restrict to the sample of respondents who are present at the time of the interview to report their own sexual orientation, that means that we must exclude people who were not home to answer the sexual orientation questions. This means that the male results may be subject to selection biases. Finally, the survey was conducted prior to the COVID-19 pandemic, and health and socioeconomic disparities may be more profound in recent years. Certainly, more population-based research is needed to better understand the sociodemographic factors and health needs of sexual minorities in Latin America. Having more large-scale studies on sexual minority health can help inform continued efforts to reduce health disparities in Latin America.

## Supporting information

S1 Fig Survey instrument visual aid. (TIF)

S1 File Chile’s health care system. (DOCX)

S2 File Survey questions for all outcomes. (DOCX)

S1 Table Prevalence of treatment for common health conditions. (DOCX)

S2 Table Main results by sexual orientation. (DOCX)
