# Supplementary figures and images for: Sexual orientation based health disparities in Chile
Source: PLoS One. 2024 Jan 25;19(1):e0296923. doi: 10.1371/journal.pone.0296923 (PMC10810431; doi:10.1371/journal.pone.0296923)

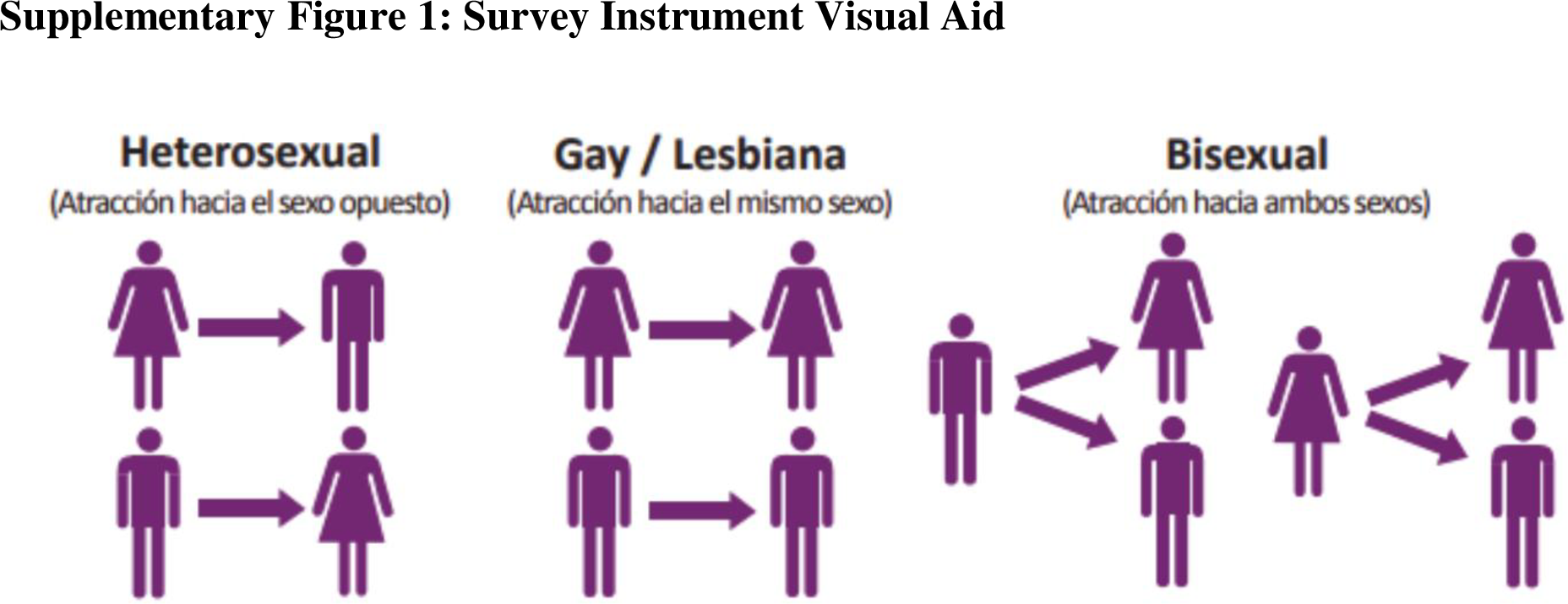

Supplement: S1 Fig — (TIF) [file pone.0296923.s001.tif]
