# Supplementary material for: Sexual orientation based health disparities in Chile
Source: PLoS One. 2024 Jan 25;19(1):e0296923. doi: 10.1371/journal.pone.0296923 (PMC10810431; doi:10.1371/journal.pone.0296923)
Supplement: S1 File — (DOCX) [file pone.0296923.s002.docx]

**Supplementary File 1: Chile’s Health Care System**

In 1979, Decree No. 2763 established Chile’s public healthcare system, *Fondo Nacional de Salud* (the National Health Fund), also called *FONASA*, which requires an employee-based payroll tax of 7% for public health insurance provided to workers and their dependent children. Payments for low-income or unemployed adults are subsidized by the national government. All Chilean nationals and non-Chileans who are legal residents and working in Chile are eligible for public healthcare. To enroll in *FONASA*, individuals can sign up online or at a *FONASA* clinic or family health center.

Individuals enrolled in *FONASA* are frequently restricted to public health clinics, facilities, and providers. In addition to *FONASA*, there also exists a private health insurance market of *Instituciones de Salud Previsional* (Social Security Institutions), also called ISAPREs, that offer healthcare through private facilities and providers. The cost of purchasing health insurance through an *ISAPRE* is differentiated by the amount of co-payments, premiums (a higher premium will confer a wider range of benefits), age, and health risk—meaning the *ISAPREs* compete for the lowest cost enrollees (i.e., young, healthy, and willing to pay additional premiums). Overall, customers pay around 9% of their taxable income. Approximately 20% of Chileans enroll in *ISAPREs*, and 75% enroll in *FONASA* (Bossert and Leisewitz, 2016)*.* The average *ISAPREs* enrollee makes more than four times the poverty level (Bossert and Leisewitz, 2016).

An additional overhaul of Chile’s health care system occurred in 2005 when President Ricardo Lagos signed into law *Acceso Universal con Garantías Explícitas* (Universal Access to Guaranteed Benefits), *AUGE* for short. *AUGE* prioritizes and requires timely medical care for 80 health conditions, including diseases and disorders that disproportionately affect sexual minorities such as HIV/AIDS, hepatitis, and depression. *AUGE* benefits are guaranteed for all enrollees regardless of their enrollment in *FONASA* or *ISAPREs*.

As a result of having a robust healthcare system, Chile has witnessed significant health improvements in recent history (e.g., decreased maternal and infant mortality rates) due to democratization, investments in public health, and improved economic opportunities.
