# Supplementary material for: Sexual orientation based health disparities in Chile
Source: PLoS One. 2024 Jan 25;19(1):e0296923. doi: 10.1371/journal.pone.0296923 (PMC10810431; doi:10.1371/journal.pone.0296923)
Supplement: S2 File — (DOCX) [file pone.0296923.s003.docx]

**Supplementary File 2: Survey Questions for all Outcomes**

We consider 5 outcomes. Below, we provide the language for each of the survey instruments we use to construct our outcome variables.

1. *Insurance status:* Respondents are asked “To what system health insurance do you belong?” Survey respondents can indicate their insurance source from one of the following options:
2. Public system – FONASA group A
3. Public system – FONASA group B
4. Public system – FONASA group C
5. Public system – FONASA group D
6. Public system – FONASA group is unknown
7. Armed Forces and Public Security Order Forces
8. ISAPRE
9. None
10. Other system

Using this variable, we create two binary variables: one for respondents who report that they do not have insurance (option *h* above) and one for respondents who report that they get their health insurance through an *ISAPRE* (option *g* above.)

1. *Health Score:* Respondents are asked “On a scale from 1 to 7, where 1 corresponds to very bad and 7 to very good, what score would give your current state of health?”
2. *Treatment for Specific Illnesses:* Respondents are asked “During the last 12 months, have you been treated for any of the following conditions? If you have been treated for more than one, choose the most important one.” The options listed are: hypertension, dental emergency, diabetes, depression, heart attack, cataracts, pulmonary disease, leukemia, asthma, gastric cancer, cervical/uterine cancer, breast cancer, testicular cancer, preventive cholecystectomy, renal failure, stroke, colorectal cancer, bipolar disorder, lupus, other health condition, I have not been treated for any health condition, and I don’t know/don’t remember. We create an indictor variable that takes value one if the respondent reports being treated for any of the listed conditions. In Appendix Table 4, we also consider each of the listed conditions as individual outcomes.
3. *Healthcare utilization:* Respondents are asked “How many doctor consultations have you had in the last 3 months?” We use the reported number of appointments to estimate effects on healthcare utilization.
