# Supplementary material for: Sexual orientation based health disparities in Chile
Source: PLoS One. 2024 Jan 25;19(1):e0296923. doi: 10.1371/journal.pone.0296923 (PMC10810431; doi:10.1371/journal.pone.0296923)
Supplement: S1 Table — (DOCX) [file pone.0296923.s004.docx]

**Supplementary Table 1: Prevalence of treatment for common health conditions**

|  | | (1) | (2) |
| --- | --- | --- | --- |
|  | | SM men | SM women |
| Hypertension | 0.003  (0.009) | 0.001  (0.011) |  |
| Dental emergency | | 0.007*  (0.004) | 0.005  (0.004) |
| Diabetes | | 0.007  (0.007) | 0.003  (0.008) |
| Depression | | 0.111**  (0.005) | 0.013  (0.009) |
| Heart attack | | -0.001  (0.001) | -0.001  (0.001) |
| Cataracts | | -0.001  (0.003) | 0.003  (0.003) |
| C.O.P.D. | | 0.004  (0.005) | 0.002  (0.002) |
| Leukemia | | -0.001  (0.001) | 0.000  (0.001) |
| Asthma | | 0.008  (0.012) | 0.004  (0.005) |
| Gastric cancer | | -0.0005**  (0.0002) | 0.000  (0.001) |
| Cervical cancer | | -  - | -0.001  (0.002) |
| Breast cancer | | -  - | 0.001  (0.003) |
| Testicular cancer | | 0.001  (0.001) | -  - |
| Prostate cancer | | 0.002  (0.003) | -  - |
| Cholecystectomy (gallbladder removal) | | -0.000  (0.000) | 0.000  (0.001) |
| Renal failure | | -0.001  (0.0006) | 0.002  (0.002) |
| Stroke | | 0.007  (0.007) | -0.001***  (0.000) |
| Colorectal cancer | | -0.001**  (0.0001) | -0.0002***  (0.000) |
| Bipolar disorder | | -0.000  (0.001) | 0.001  (0.002) |
| Lupus | | 0.000  (0.001) | -0.002***  (0.000) |
| Other condition | | 0.053***  (0.017) | -0.011  (0.011) |
|  | |  |  |
| N | | 72,660 | 127,030 |

Notes: *, **, and *** denote statistical significance at 10%, 5%, and 1%, respectively. Standard errors are reported below estimates in parentheses. OLS models. Specifications control for age and its square, indigenous and immigrant status, gender minority status, education, marital status, the number of adults and number of children in the household, urbanicity, survey year, and region. Results use person-level survey weights, and standard errors are robust to heteroskedasticity.
