# Supplementary material for: Sexual orientation based health disparities in Chile
Source: PLoS One. 2024 Jan 25;19(1):e0296923. doi: 10.1371/journal.pone.0296923 (PMC10810431; doi:10.1371/journal.pone.0296923)
Supplement: S2 Table — (DOCX) [file pone.0296923.s005.docx]

**Supplementary Table 2: Main results by sexual orientation**

|  | | (1) | (2) | (3) | (4) | (5) |
| --- | --- | --- | --- | --- | --- | --- |
|  | | Uninsured | Private Insurance | Health  score ≥5 | Treated for common illness in last 12 months | Number of primary care consultations in the last 3 months |
| **Panel A: Men** | | | | |  |  |
| Gay/Lesbian | -0.014 | 0.019 | -0.027* | 0.105*** | 0.173*** |  |
|  | (0.012) | (0.024) | (0.015) | (0.026) | (0.066) |  |
| Bisexual | -0.007 | 0.173** | -0.027 | 0.074 | 0.169 |  |
|  | (0.019) | (0.063) | (0.018) | (0.060) | (0.172) |  |
| Mean of outcome: | | 0.039 | 0.195 | 0.842 | 0.313 | 0.306 |
| N | 67,029 | 67,029 | 67,740 | 67,552 | 67,866 |  |
| **Panel B: Women** | | | |  |  |  |
| Gay/Lesbian | | 0.015 | -0.017 | -0.029 | 0.005 | 0.054 |
|  | | (0.011) | (0.017) | (0.019) | (0.022) | (0.077) |
| Bisexual | | 0.033 | 0.043 | -0.048* | 0.043 | 0.072 |
|  | | (0.021) | (0.043) | (0.026) | (0.043) | (0.085) |
| Mean of outcome: | | 0.022 | 0.119 | 0.796 | 0.418 | 0.431 |
| N | | 122,349 | 122,349 | 122,907 | 122,648 | 123,100 |

Notes: *, **, and *** denote statistical significance at 10%, 5%, and 1%, respectively. Standard errors are reported below estimates in parentheses. OLS models. Specifications control for age and its square, indigenous and immigrant status, gender minority status, education, marital status, the number of adults and number of children in the household, urbanicity, survey year, and region. Results use person-level survey weights, and standard errors are robust to heteroskedasticity.
